# Supplementary material for: Reading Profiles in Multi-Site Data With Missingness
Source: Front Psychol. 2018 May 8;9:644. doi: 10.3389/fpsyg.2018.00644 (PMC5952106; doi:10.3389/fpsyg.2018.00644)
Supplement: Supplementary file 1 [file Image_1.pdf]

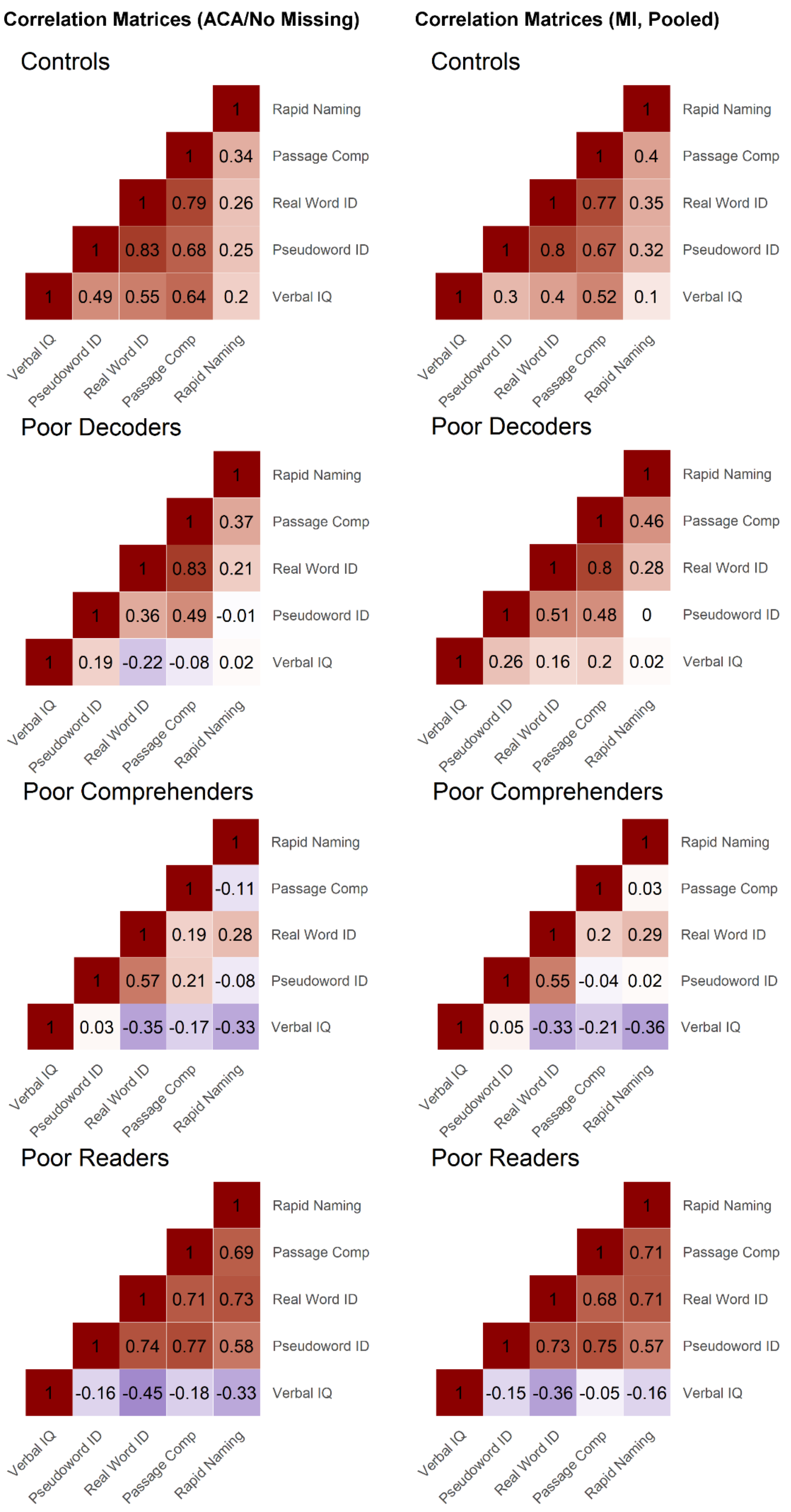

Supplemental Figure 1. Correlation matrices demonstrate different patterns of behavioral measure covariance across the reading profiles. These results are consistent across the cases with no missing values (N = 327; left column) and when cases with imputed data are included (N = 726; right column; pooled results across 10 imputations; excluding the 198 cases in the reading profile training dataset). The Control subjects demonstrated positive associations between all the test scores, with moderate to strong associations among the Word Attack, Word ID, and Passage Comprehension test scores (top row). The Poor Decoders demonstrated positive relationships between most of the reading test scores, and Verbal IQ weakly related to the other measures. The Poor Comprehenders and Poor Readers both showed negative correlations between Verbal IQ and the other measures. The Poor Readers demonstrated stronger positive correlations between most of the other test scores, which differed from the Poor Comprehenders. An identical color scale is used for each of the correlation matrices, with red for positive correlations and blue for negative correlations, which are also printed in each of the cells.
